# MILD: Minimizing Idle Listening Energy Consumption via Down-Clocking for Energy-Efficient Wi-Fi Communications †

**DOI:** 10.3390/s25041155

**Published:** 2025-02-13

**Authors:** Jae-Hyeon Park, Young-Joo Suh, Dongdeok Kim, Harim Lee, Hyeongtae Ahn, Young Deok Park

**Affiliations:** 1 Department of Computer Science and Engineering, Pohang University of Science and Technology (POSTECH), 77 Cheongam-ro, Nam-gu, Pohang 37673, Republic of Korea; parkeric3@postech.ac.kr (J.-H.P.); dongdokee@postech.ac.kr (D.K.); 2 Graduate School of Artificial Intelligence, Pohang University of Science and Technology (POSTECH), 77 Cheongam-ro, Nam-gu, Pohang 37673, Republic of Korea; yjsuh@postech.ac.kr; 3 Department of Electronic Engineering, Kumoh National Institute of Technology, 61 Daehak-ro, Gumi 39177, Republic of Korea; hrlee@kumoh.ac.kr; 4 Department of Computer Engineering, Kumoh National Institute of Technology, 61 Daehak-ro, Gumi 39177, Republic of Korea; htahn@kumoh.ac.kr; 5 Department of Computer Engineering, Yeungnam University, 280 Daehak-ro, Gyeongsan 38541, Republic of Korea

**Keywords:** wireless communications, energy efficiency, idle listening adapting clock rate, OFDM subcarrier, DFS

## Abstract

Mobile devices, such as smartphones and laptops, face energy consumption challenges due to battery limitations, with Wi-Fi being one of the major sources of energy consumption in these devices. The IEEE 802.11 standard addresses this issue with Power Saving Mode (PSM), which reduces power consumption but increases latency. To mitigate this latency, Adaptive-PSM (A-PSM) dynamically switches between PSM and Constantly Awake Mode (CAM); however, the associated Idle Listening (IL) process still results in high energy consumption. Various strategies have been proposed to optimize IL time; however, Medium Access Control (MAC)-level contention and network delays limit their effectiveness. To overcome these limitations, we propose MILD (Minimizing Idle Listening energy consumption via Down-clocking), a novel scheme that reduces energy consumption without compromising throughput. MILD introduces specialized preambles for Packet Arrival Detection (PAD) and Device Address Recognition (DAR), allowing the client to operate in a down-clocked state during IL and switch to full clocking only when necessary. Experimental results demonstrate that MILD reduces energy consumption by up to 23.6% while maintaining a minimal throughput loss of 12.5%, outperforming existing schemes.

## 1. Introduction

The energy efficiency of mobile devices such as smartphones and tablet PCs is a significant issue due to their limited battery life. Recent studies have shown that when communicating with a common 802.11 wireless Local Area Network (LAN) card, the battery can deplete within a few hours [1,2]. In mobile devices, Wi-Fi remains active, even during screen-off periods and low CPU/GPU activity to maintain network responsiveness. However, this can lead to considerable energy consumption. As users frequently have screens off for extended durations, Wi-Fi energy usage can become a significant contributor in such instances. For instance, Xia et al. [3] report that Wi-Fi energy drain during screen-off periods may account for between 29% and 58% of total energy usage, while Pramanik et al. [4] estimate that Wi-Fi may consume approximately 25% of total energy when factoring in overall application operation on a smartphone. This issue is further accentuated in Internet of Things (IoT) devices, where reduced CPU/GPU demands mean that Wi-Fi-related energy costs can comprise a more substantial fraction of total energy consumption [5].

To reduce energy consumption, the IEEE 802.11 standard includes Power Saving Mode (PSM) [6], in which devices remain in a sleep state during inactive periods, significantly reducing power usage. However, PSM’s energy savings come at the cost of increased latency, particularly for interactive applications. Adaptive Power Saving Mode (A-PSM) dynamically switches between PSM and Constantly Awake Mode (CAM), balancing energy savings with responsiveness. Yet, the energy wasted during Idle Listening (IL) in A-PSM remains a persistent issue, especially under high contention or unpredictable traffic conditions.

Given this tradeoff, prior A-PSM studies have focused on optimizing IL time. Various approaches dynamically adjust IL time, such as isolating traffic to avoid peak periods [7] or using machine learning to classify traffic [8]. However, Medium Access Control (MAC)-level contention and network-level delays limit further IL time reduction, making optimization difficult [9]. Additionally, the recent IEEE 802.11 standards have introduced Automatic Power Save Delivery (APSD) [6], Target Wake Time (TWT) [10] and Restricted Access Window (RAW) [11] to improve energy efficiency. However, these mechanisms have limited backward compatibility in real-world applications, and their adoption rate remains relatively low [12], which constitutes a limitation. Moreover, IL time still persists in the power saving MAC operations of recent standards [13,14,15].

To address the limitations of A-PSM, the down-clocking method (also called dynamic frequency scaling, or DFS) is introduced to reduce power consumption by dynamically lowering the device’s clock rate [9,16,17,18]. However, approaches such as [16,17] result in lower data rates due to the use of low-order modulation. In contrast, E-MiLi [9] switches to full-clock during data decoding, maintaining data rates while reducing power consumption during idle listening periods.

**Figure 1:**
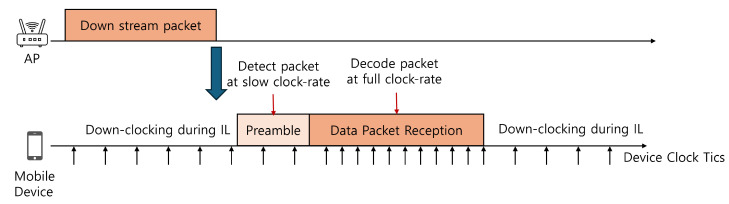
Example of down-clocking operation in a downstream scenario.

Figure 1 illustrates the operation of the down-clocking methods such as E-MiLi in a downstream scenario. During idle listening (IL), the client cannot decode packets in the down-clocking state and must restore its full clock rate to process incoming data. Standard preambles cannot be decoded in this state either; therefore, Access Point (AP) attaches a special preamble for packet detection and address recognition. The client uses auto-correlation on this preamble to detect packet arrival and recognize the device address even when down-clocked. After detecting the packet arrival and confirming the address match, the device switches to full-clocking to decode the packet before returning to the down-clocking state during subsequent IL periods.

**Figure 2:**
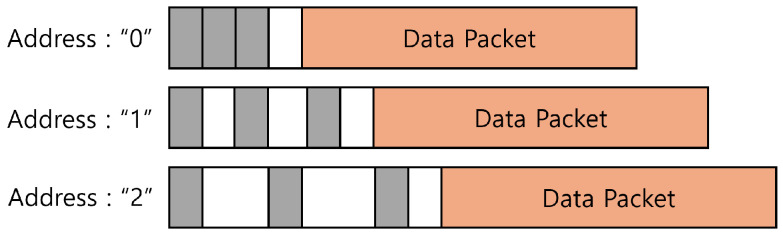
Address overhead of down clocking.

This approach offers greater energy savings compared to many A-PSM techniques; however, it presents two notable limitations. First, the special preamble required for down-clocking reduces throughput as it consumes more channel time. As the number of clients increases, the preamble length grows, resulting in inefficiencies in E-MiLi. Figure 2 illustrates the addressing overhead caused by address representation. To handle this problem, address sharing is introduced, enabling multiple clients to use the same address and reducing the overhead from long preambles. Second, while address sharing reduces overhead, it may result in fault wake-ups. In this case, clients may incorrectly process unintended packets, which leads to energy waste. This creates a trade-off: increasing the address sharing factor (i.e., more clients sharing the same address) reduces the occurrence of fault wake-ups and saves energy but degrades throughput. On the other hand, lowering the sharing factor improves throughput but increases energy waste due to more frequent wake-ups.

To address these limitations, MILD introduces a novel preamble design with two key features: (1) a fixed, short preamble length, and (2) the ability to encode a large number of addresses without address sharing. The preamble comprises Packet Arrival Detection (PAD) preamble followed by Device Address Recognition (DAR) preamble. We optimized the PAD preamble length through Universal Software Radio Peripheral (USRP) experiments to improve the success rate of auto-correlation-based packet detection. For the DAR preamble, we leverage the subcarriers of an Orthogonal Frequency-Division Multiplexing (OFDM) symbol to encode client addresses. Each subcarrier is used to represent a single bit, allowing the address to be encoded as multiple bits based on the number of available subcarriers. Unlike previous methods, MILD maintains a constant preamble length regardless of the number of clients connected to the AP, avoiding throughput degradation. Additionally, MILD’s address encoding minimizes false clock recovery, reducing unnecessary energy consumption.

We validate the feasibility of MILD through experiments on the USRP/GNURadio platform [19] and conduct simulation-based performance evaluations. The simulation results show that MILD provides significantly lower energy consumption and higher network throughput compared with existing schemes.

## 2. Related Work

### 2.1. Wi-Fi MAC Rules for Energy Saving

Wi-Fi chipsets typically support multiple modes, each with varying energy consumption levels. Most commercial Wi-Fi chipsets switch between two power modes: a high-power CAM, which consumes high energy even when idle, and an energy-conserving PSM [6] with low power consumption but no communication capabilities. In CAM mode, the Wireless Network Interface Card (WNIC) is always awake. It operates with the best performance and least delay, but stays awake for the entire duration, resulting in high energy consumption.

In the PSM scheme, the WNIC wakes up periodically to receive data. When a new packet arrives that is not part of the buffered packets, it is delayed until the next beacon period (typically 100 ms). While this approach works for power saving, it adds packet delay caused by the buffering of packets during the beacon intervals. To overcome this problem, Wi-Fi chipset vendors have adopted adaptive PSM, where the Wi-Fi interface remains idle for additional data packets in the IL state for a certain period after receiving data. Recently, there have been several alternatives to PSM. Most deal with switching between the active mode and PSM. The active mode requires the Wi-Fi radio to remain active, requiring significantly more power.

**Static PSM** was defined by the IEEE 802.11 PHY/MAC standard. The AP maintains PSM queues for each Mobile Node (MN). The AP buffers incoming packets from servers and maps their address into Traffic Indication Map (TIM) fields in the beacon. The AP indicates the presence of the buffered data frames via beacon frames. The MN periodically wakes up to listen to a beacon frame from the AP. If the MN A learns that the AP has data frames for it, it wakes up the entire radio and sends the PS-POLL to obtain the data frame. The client wakes up periodically to receive the beacon, and if its device address is set in the TIM field, it sends a PS-POLL message to the AP. The AP sends the buffered packet after receiving the PS-POLL message. The client device switches to sleep mode after receiving the data packet, but if the MORE bit is present in the data frame, the MN sends the PS-POLL frame again to request additional buffered data frames. If the MORE-DATA field is unset (0), which is in the data frame, the MN goes to sleep and remains in sleep mode until the next beacon listening time. However, because the client device cannot receive data packets during sleep mode, the data packets are delayed until the next beacon period. Therefore, network throughput is degraded.

**A-PSM** tried to solve the delayed packet problem. The client device switches between CAM and PSM based on heuristics results. The device notifies the AP of whether it is in PSM or CAM state by sending NULL data frames with the power management bit set to 1 or 0. The client wakes up to receive data packets and switches to the CAM state, which extends for a certain amount of time, called the tail time, after receiving the data packets. The CAM state transitions to the PSM state when the tail time is expired. The tail time is adjusted based on heuristics. However, since the device remains in the CAM state even when no data are available, Idle Listening (IL) time occurs. A-PSM can reduce power consumption according to the optimization of tail time scheduling.

Several studies have attempted to solve the scheduling problem. NAPman leverages AP virtualization and an energy-aware fair scheduling algorithm to minimize client energy consumption and reduce unnecessary retransmissions [20]. NAPman aims to deliver energy savings without unfairness. Sleepwell is system that achieves energy efficiency by evading network contention [7]. However, the underlying trade-off between network throughput and energy consumption remains a challenge in Wi-Fi research. Snooze dynamically coordinates client sleep patterns by adapting to traffic bursts and link quality changes [21]. As another approach, an energy-efficient algorithm using data offloading based on traffic characteristics, has been introduced [22]. Network traffic with moderate data transfer speeds will either suffer from high latency or consume extra power. Therefore, it is logical to investigate the use of other schemes to manage the power budget with wireless communication.

**Automatic Power Save Delivery (APSD)**, introduced in the IEEE 802.11e amendment [6], is a power-saving mechanism primarily designed to enhance Quality of Service (QoS) in wireless networks. APSD operates in two modes: scheduled (S-APSD) and unscheduled (U-APSD). In S-APSD, transmissions are scheduled at specific intervals, allowing the station (STA) to wake up only at predefined times to receive data, whereas in U-APSD, the STA sends a trigger frame to request pending frames from the AP as needed. This mechanism is particularly suited to multimedia applications with stringent delay requirements, such as voice over IP, as it combines energy efficiency with QoS.

**Target Wake Time (TWT)**, introduced in IEEE 802.11ax (Wi-Fi 6) [10], is a power-saving feature that operates on the Orthogonal Frequency Division Multiple Access (OFDMA) mechanism, allowing STAs to access specific resource units (RUs) at scheduled wake times. Through mechanisms like trigger-enabled TWT, TWT-requesting STAs can utilize upstream OFDMA transmissions to either signal the AP for pending frames or send data frames. The AP, in turn, allocates precise RUs for both upstream and downstream transmissions, enabling efficient, simultaneous data exchange for multiple STAs. This approach minimizes contention and enhances energy efficiency, particularly in environments with sufficient STAs to fully leverage OFDMA.

**Restricted Access Window (RAW)**, introduced in IEEE 802.11ah (Wi-fi HaLow) [11], is a power-saving mechanism designed to structure upstream traffic by allocating specific time slots (RAW slots) for groups of STAs. During their assigned slots, STAs can transmit without interference from other groups while being restricted from transmitting during other slots. The AP dynamically schedules RAW slots and subslots based on consecutive association IDs and announces group configurations in each beacon interval. This method reduces contention and improves energy efficiency by enforcing scheduled access, while the use of RAW backoff during slots ensures optimal use of the medium.

While features like APSD, TWT, and RAW introduce significant advancements in energy efficiency and performance, their limitations in backward compatibility hinder widespread adoption. According to Wi-Fi Alliance certification programs, MAC-layer energy-saving features are categorized as follows: U-APSD is included in the Wi-Fi Multimedia (WMM)-Power Save certification, TWT in the Wi-Fi 6 certification, and RAW in the Wi-Fi HaLow certification. However, as shown in Table 1, the adoption rates remain inconsistent across device categories [12]. For example, WMM-Power Save is adopted by 68% of smartphones but has an adoption rate of 0% for printers and smart home devices, and only 7% for TVs. Similarly, Wi-Fi 6 exhibits an adoption rate of merely 4% for smartphones, with even lower rates for printers( 0%), smart home devices (0%), and TVs (1%). Wi-Fi HaLow adoption currently stands at 0% across all applications. Considering these observations, the widespread utilization of legacy PSM by off-the-shelf devices underscores its importance. Enhancing legacy PSM offers a generally viable solution, leveraging its extensive deployment and compatibility with existing devices.

In addition, APSD, TWT, and RAW encounter significant energy consumption challenges attributed to IL, similar to those observed in A-PSM. Pérez-Costa et al. [13] identified specific scenarios in APSD where IL arises, including synchronization issues and the excessive use of QoS null frames. Yang et al. [14] investigated cases in TWT where improper scheduling or suboptimal wake/doze period negotiation led to increased IL and energy consumption. Similarly, Kureev et al. [15] analyzed RAW conditions, demonstrating how poorly allocated time windows or device contention exacerbate IL. Notably, RAW, designed to complement A-PSM, introduces additional IL due to the tail time inherent to A-PSM operations. These studies emphasize that IL remains a persistent limitation across power-saving mechanisms, requiring further optimization to mitigate the impact of IL.

**Figure 3:**
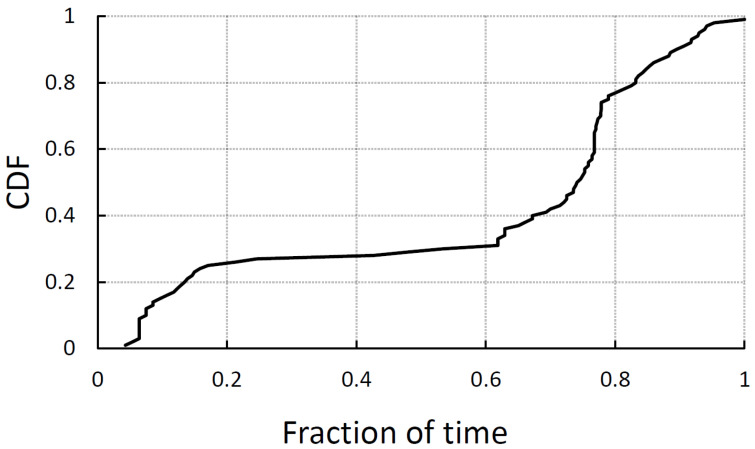
Normalized fraction of time spent in idle listening state across various traffic patterns and environments.

For this reason, Idle Listening (IL) still constitutes a significant portion of the total transmission time in commercial devices. To demonstrate this, we collected Wi-Fi packet traces directly from a university library in 2024. The tools used for data collection include Wireshark and AirPcap, and data were gathered over a one-week period. Furthermore, to conduct a more detailed analysis of each client’s active time, we segregated the operational states into transmission (Tx)/ reception (Rx), sleep, and IL. This detailed segmentation allows for a more precise understanding of how clients distribute their time among different states, emphasizing that IL constitutes a significant fraction of overall network activity. Figure 3 presents the normalized proportion of time spent in the IL state. In the university library environment, over 70% of clients exhibit an IL ratio exceeding 0.55. These results indicate that legacy PSM schemes and recent power-saving mechanisms relying on MAC operations face fundamental limitations in reducing IL duration due to MAC-layer contention, periodic management frames, and network-level delays. Additionally, legacy PSM schemes perform inefficiently in mixed-traffic environments, where various traffic types coexist, further exacerbating IL-related inefficiencies.

### 2.2. Extremely Low-Power Communication Technologies for IoT and Sensor Networks

Various technologies have been researched and developed to achieve extremely low power consumption in IoT and sensor network environments. These technologies are designed to extend the battery life of devices and maintain network connectivity even in low-power states. Efforts to minimize power consumption while enabling periodic data transmission and monitoring functions for IoT devices are steadily increasing.

**Wake-Up Receivers (WUR)**, defined in the IEEE 802.11ba standard, represent a prominent low-power technology [23]. WUR allows devices to remain in a low-power state and only consume power when data need to be received. Blobel et al. [24] proposed a wake-up WLAN system that extends standard IEEE 802.11 by incorporating wake-up signals for energy-efficient communication, using selective wake-up receivers to enable low-latency, low-power connectivity in IoT environments. While this design reduces unnecessary power usage, WUR suffers from limitations, such as low data transmission rates and limited adoption in commercial products. These constraints pose challenges for the widespread use of WUR in practical applications.

**Wi-Fi backscatter**, another approach, enables extremely low-power communication by reflecting existing signals to transmit data [25]. Yuan, L. et al. [26] present an ultra-low-power system that enables battery-free IoT backscatter communication by leveraging multiple excitation signals such as Wi-Fi, Bluetooth, and ZigBee, providing high identification accuracy and stable data transmission. Since backscatter technology utilizes existing signals without generating new ones, it is advantageous in terms of energy efficiency. However, it requires additional hardware, and its low data transmission rate makes it unsuitable for real-time data transfer or large-scale data transmission. Consequently, while Wi-Fi backscatter excels in power efficiency, it faces clear limitations in terms of communication performance.

These technologies often face challenges in achieving widespread adoption due to compatibility issues with existing infrastructure and additional hardware requirements. While these methods effectively contribute to reducing energy consumption, their applicability is often limited to specific-purpose applications or constrained environments. A solution targeting general data communication must meet the requirements for higher data transmission rates, compatibility with existing infrastructure, and reduced hardware dependency.

### 2.3. Down-Clocking Method

The down-clocking scheme focuses on a different aspect of energy efficiency. Since IL time cannot be reduced any further due to Wi-Fi’s CSMA, the down-clocking scheme reduces energy consumption by placing the radio into a down-clocked state rather than using IL scheduling in PSM. Instead of IL scheduling in PSM, the down-clocking scheme put the radio in the down-clocking state to reduce energy consumption. E-MiLi utilizes the down-clocking state during IL and switches between full-clocking and down-clocking depending on when the client needs to receive data. In this scheme, when the client only needs to perform Clear Channel Assessment (CCA) without receiving data, it keeps the clock rate low. The client controls the radio clock rate on a fine-grained, per-packet basis to reduce energy consumption during IL. It opportunistically lowers the clock rate during IL and restores it to full speed before transmitting or after detecting a packet. When a packet arrives, the client continuously monitors the energy level of a sequence that corresponds to the packet’s preamble length. It calculates a correlation output and determines whether the data it needs to receive have arrived when the energy level exceeds a certain threshold. Upon detecting incoming data, the client recovers the full clock rate and receives the data. Unlike A-PSM, this scheme operates with low power during the idle listening state when there is no need to receive data, significantly reducing the energy wasted during IL and thereby reducing overall energy consumption. Therefore the energy wasted in the IL state is fundamentally reduced, thereby reducing energy consumption linearly.

However, if the packet is at a lower clock rate than required, the packet cannot be decoded. According to Nyquist sampling theory, the receiver’s sampling rate needs to be at least twice the bandwidth of the transmitted signal to decode the packet. To receive packets, the client must restore to full-clock. For this restoration process, E-MiLi adopts a long preamble design, which decreases network throughput [9]. As a result, it was possible to obtain high energy efficiency, but it sacrificed overall network throughput in an environment with a high number of clients.

In addition to this, there have been studies performing communication with down-clocking in various ways. SloMo is a transceiver system design based on compressive sensing, which allows data packets to be decoded during the down-clocked state, but it does not primarily focus on energy efficiency [17]. Gap-sense designed preambles using signal correlation during the down-clocking state for heterogeneous device communication but it was not related to energy consumption [27]. Technologies like SloMo and Gap-Sense originate from foundational studies and have since evolved into the broader field of Cross-Technology Communication (CTC). BlueFi [28] enables communication from Bluetooth to Wi-Fi by utilizing the Wi-Fi chipset’s spectral analysis capability to detect and interpret BLE signals, achieving high-speed, bidirectional transmission. TransFi [29] emulates various wireless standards on commodity Wi-Fi devices by manipulating MAC payloads and MIMO streams, allowing for custom PHY functionality without hardware modifications. These methods explore interoperability across differing clock rates; however, the reduction in data rate that results from decoding at lower clock speeds remains a fundamental limitation of CTC. Additionally, these techniques fall outside the scope of our study, as they do not primarily address energy efficiency, a central focus of our research. LiteNap [16] improves LoRa energy efficiency by enabling downclocked ’light sleep’ mode for packet reception, addressing frequency aliasing issues caused by undersampling through phase jitter and frequency leakage detection. However, it does not concern address identification in dense LoRa environments.

Although there are various studies on down-clocking methods, it is important to consider the trade-off between energy efficiency and throughput. Our PHY-aided MAC approach uniquely addresses both interoperability and energy efficiency without a trade-off in throughput, making it a distinctive solution in this area.

### 2.4. PHY-Aided MAC Approach

One of the main reasons for the low utilization of MAC operations is the exchange of coordination packets between the AP and the client. The PHY-aided MAC approach can overcome significant performance barriers [30]. The feasibility of programming the PHY layer through software-defined radio (Virtex-IV) to improve MAC performance has already been demonstrated [31].

Several studies have shown that MAC performance can be improved using information obtained through the PHY-aided MAC approach. For instance, DOMINO leverages OFDM polling to obtain queue status from clients [32]. RAMCAST enables multiple multicast clients to transmit feedback to AP simultaneously without collision [33]. Several other PHY-aided approaches have been explored in terms of security, throughput, and QoS, but few have focused on energy efficiency [34,35].

However, our approach to addressing energy consumption with the PHY-aided MAC approach is unique. PHYCAST is an energy-saving scheme based on overhearing, but it does not overcome the fundamental limitations of sleep scheduling [36]. The physical information obtained from the PHY layer can be measured robustly even when the device clock is low. We utilize the energy level of the measurable OFDM subcarriers, even when the clock is low.

## 3. Validation of Proposed Method

### 3.1. Simulation and Testbed Experimental Setup

To validate the feasibility and performance of the proposed MILD scheme, we utilized the USRP/GNURadio platform to conduct both experimental and simulation-based evaluations. This section outlines the materials and methods used in these validations.

**Hardware and software setup**: We configured the USRP N210 hardware to transmit and receive 802.11a/g OFDM symbols. E-MiLi utilizes a non-standard preamble format to support address recognition in down-clocking, adjusting the preamble length to represent addresses. The proposed method ensures backward compatibility by modifying legacy A-PSM. Based on this design choice, 802.11a/g is selected, as it provides a preamble structure applicable to generic standards. It is challenging to extend our implementation beyond 802.11a/g due to its preamble structure constraints; thus, our experiments focus on these standards. The client device is programmed to operate in a down-clocked state, detecting packets using the proposed PAD and DAR mechanisms. The experiments are conducted on a 20 MHz channel, with the clock rate adjusted according to the down-clocking factor.

**Packet detection accuracy measurement**: To evaluate packet detection accuracy, we vary the PAD preamble length and measure the detection success rate with different down-clocking factors. As referenced by Thomson et al. [37], an SNR of 10 dB is typically regarded as the minimum threshold for reliable Wi-Fi data reception. Based on this criterion, communication at SNR levels below 10 dB becomes challenging, even under standard conditions. In line with the 802.11 standard, the minimum input sensitivity for BPSK (MCS0) at a 20 MHz bandwidth is defined as –82 dBm [6]. Given a typical noise level of –95 dBm, this corresponds to an SNR of 13 dB, which sets a foundational threshold for reliable Wi-Fi communication [38]. Furthermore, in real-world environments, signal strengths typically range from –70 dBm to –45 dBm, translating to SNR levels between 25 and 50 dB [39]. Thus, based on the proposed method’s robust performance above these baseline SNR levels, it demonstrates high suitability and feasibility for practical applications, aligning with typical environmental requirements for stable Wi-Fi communication. Using short OFDM-based training symbols, we incrementally increase the number of symbols in the PAD preamble. The detection accuracy reaches about 99% within 8 μs for a down-clocking factor of 2, with similar results for higher factors.

**Device address recognition accuracy measurement**: The DAR preamble is tested using 52 OFDM subcarriers, with different subcarriers encoding the address bits. We measure the false negative probability by testing the client’s ability to correctly recognize its address at various SNR levels. The address recognition accuracy remains above 99% for SNR values greater than 10 dB.

**Simulation-based performance evaluation**: We conduct network simulations using the OPNET simulator to assess the throughput and energy consumption of MILD under various traffic conditions. The simulation is conducted in an environment where up to 50 clients are connected to a single AP. For the throughput evaluation, traffic is generated under saturated conditions. For the energy consumption evaluation, a traffic generator is created by profiling various traffic patterns, which are then used in the simulation. MILD’s performance is compared to traditional A-PSM and E-MiLi schemes, both with and without address sharing. MILD maintains a constant preamble length, resulting in negligible throughput loss, while E-MiLi experiences significant degradation as the number of clients increases.

Through these evaluations, we demonstrate that MILD significantly reduces energy consumption without compromising network throughput, even in environments with high client density and varying traffic patterns.

### 3.2. Operation of the Proposed Method

**Figure 4:**
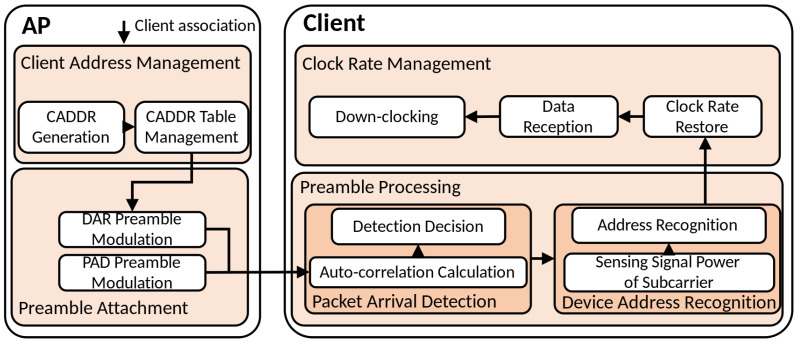
Operation flow of MILD.

The core process of the MILD technique involves the client adjusting its clock rate dynamically during data reception. While during IL, the client operates in a down-clocked state and uses the Packet Arrival Detection (PAD) mechanism to detect incoming packets. Once PAD is triggered, the client switches to the full clock rate through the Packet Address Recognition (DAR) process, which verifies whether the packet’s destination matches the client’s unique address, referred to as the Client Address (CADDR). During data transmission, the client follows the standard 802.11 MAC protocol. Figure 4 illustrates the process by which the client switches clock states during packet reception, using both PAD and DAR mechanisms.

To support the client’s PAD and DAR processes, MILD requires specific AP operations. The AP generates a PAD preamble composed of short training symbols to assist with packet detection. Furthermore, during the association phase, the AP creates and maintains a CADDR table shared with the client. The CADDR is encoded into the DAR preamble using the subcarriers of an OFDM symbol, allowing the client to match and recognize its address. When transmitting data, the AP modulates and attaches both the PAD and DAR preambles to the packet to ensure packet detection and address recognition by the client.

In the PAD process, the client computes an auto-correlation value using the short training symbols in the PAD preamble, similar to other packet detection algorithms [27,40]. However, in the down-clocked state, due to the reduced number of samples for auto-correlation, the detection time and threshold values for reliable detection become more restrictive and must be determined experimentally. When the auto-correlation value exceeds the experimentally defined threshold, the client detects the packet’s arrival.

For the DAR process, the AP encodes the client’s address onto OFDM subcarriers using on–off keying. Binary ‘0’ is mapped to zero amplitude, and binary ‘1’ is mapped to a random complex number. The client detects the energy levels in the DAR preamble to distinguish between ‘0’ and ‘1’, identifying the address. Once the address is verified, the client remains in full clocking to receive the data and returns to the down-clocking state during idle periods.

**Figure 5:**
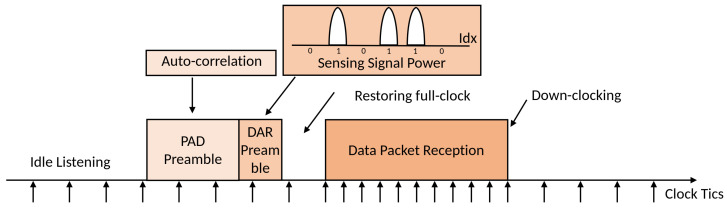
Example illustrating the client’s operation in MILD.

For a clearer understanding of the DAR operation, consider the following example. As shown in Figure 5, assume the destination CADDR of the packet is “22”, and the client’s CADDR is also “22”. For simplicity, we assume the DAR preamble uses six subcarriers (a more detailed discussion on the number of subcarriers will be covered in Section 3.4). The AP modulates the DAR preamble to represent “22” by generating signals in the 1st, 3rd, and 4th subcarriers. The client detects positive signal energy levels in the active subcarriers and confirms that the CADDR is “010110” (i.e., “22”). If the CADDR matches, the client switches to full clocking to receive the data.

In the next section, we will discuss the preamble design and the technical challenges addressed for reliable PAD and DAR operation in the down-clocking state.

### 3.3. Design of PAD Preamble

We use the auto-correlation approach for packet detection, sensing the short training field that contains the OFDM symbol, similar to other studies [41]. We apply the same algorithm as the default preamble, utilizing short training symbols of the OFDM scheme for correlation in packet detection. There are 16 time-domain samples corresponding to one sequence when sampling at the full-clock rate in 20 the MHz bandwidth. Let us denote the time-domain samples as *r* (·). The following is the auto-correlation output:

(1) $$auto\left(i\right)=\sum_{j=0}^{15}r\left(i+j\right)r\left(i+j+16\right)$$

When index *i* is within the range of the short preamble, the output of Equation (1) is maintained at a high value [42]. Therefore, peaks are clearly detected when the appropriate time passes. The client detects the packet arrival when the average output of Equation (1) exceeds an experimentally determined threshold.

According to Nyquist sampling theory, to successfully decode a signal, the client must have a sampling rate $F_{s}$ at of least twice the Frequency Bandwidth (BW) of the transmitter. Otherwise, signal aliasing occurs, causing signals with different frequencies to fold into the same frequency band. Since the clock rate of the client in MILD is down-clocked, signal aliasing will occur as follows. If the client radio operates at $F_{s}=\frac{BW}{D}$, where *D* is a down-clocking factor, the original signal bandwidth will be aliased into $\left[-\frac{BW}{2D},\frac{BW}{2D}\right]$. A frequency $f=n\frac{BW}{D}+f_{\mathrm{aliased}}\;\left(n=1,2,\ldots ,D-1\right)$ folds into $f_{\mathrm{aliased}}$ [16]. Thus, Equation (1) holds when r(i)=r(i+8) or r(i)=r(i+4) for down-clocking factors of D=2 or D=4, respectively. As a result, ambiguity arises where the client cannot distinguish the folded samples. As the down-clocking factor increases, the valid time-domain samples used for the auto-correlation calculations decrease proportionally, resulting in reduced packet detection accuracy.

Algorithm 1 describes the DAR process on the client side. We provide an explanation of the detailed process. The client receives a time-series signal, r(i), during the IL period (line 1). Initially, key parameters such as the down-clocking factor *D*, an experimentally determined threshold, and the variable for auto-correlation output (auto_corr), are initialized. The initial value of auto_corr is set to 0. Additionally, the sample length (sample_length) and the correlation window size (correlation_window) are set to 16 and 15, respectively (lines 2–6).

During the IL period, the client shifts the correlation window and repeatedly calculates the auto-correlation output, auto_corr(i). In the down-clocking state, the signal becomes aliased, so auto_corr(i) is calculated by summing the products of r(i+j) and $r\left(i+j+\frac{\mathrm{sample}\_\mathrm{length}}{D}\right)$, instead of r(i+j+sample_length), across the correlation window. This summation evaluates the similarity between shifted sequences of the time-domain samples and is expressed as follows (lines 7–8):

(2) $$\mathrm{auto}\_\mathrm{corr}\left(i\right)=\sum_{j=0}^{\mathrm{correlation}\_\mathrm{window}}r\left(i+j\right)\cdot r\left(i+j+\frac{\mathrm{sample}\_\mathrm{length}}{D}\right).$$

When the current correlation output (auto_corr) exceeds the predefined threshold, the client determines that the PAD preamble for packet detection, which is sent by the AP, has been received. In this case, the client concludes that a packet is detected, exits the PAD process, and proceeds to the DAR process (lines 9–12). If the auto-correlation value does not exceed the threshold during the IL period, the loop for auto_corr calculation continues. After the IL period ends, the client concludes that no packet is detected (line 14). By leveraging this sequential process of auto-correlation and threshold comparison, Algorithm 1 efficiently identifies packets in the input time-domain samples under down-clocked conditions.
**Algorithm 1** PAD Process for Packet Detection in MILD1:**Input:** r(i) ▹Time-domain samples2:**Parameters:**  *D*▹Down-clocking factor3:**Threshold:** threshold▹Experimentally determined threshold4:auto_corr←0▹Initialize auto-correlation output5:sample_length←16▹Length of time-domain samples for one sequence6:correlation_window←15▹Window size for auto-correlation calculation7:**for** i=0 to N−correlation_window
**do**8:   *aut*$o\_corr\left(i\right)\leftarrow \sum_{j=0}^{correlation\_window}r\left(i+j\right)\cdot r\left(i+j+sample\_length/D\right)$9:   **if** auto_corr(i)>threshold
**then**10:    **Packet Detected**11:    **Exit**12:   **end if**13:**end for**14:**Return: No Packet Detected**

**Figure 6:**
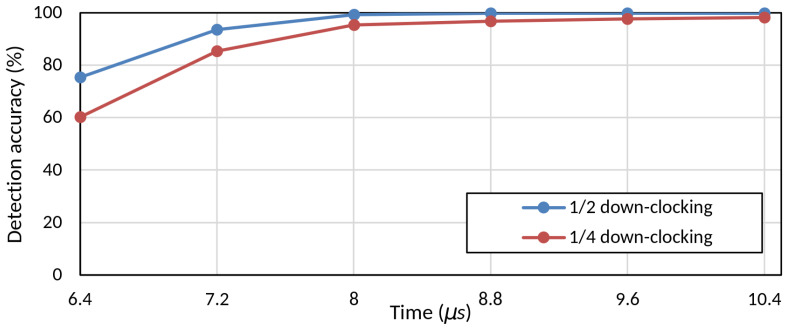
PAD accuracy results with USRP testbed.

We experimentally determined the minimum number of time-domain samples required for auto-correlation to achieve high packet detection accuracy in the down-clocking state. For this purpose, we constructed a USRP/GNURadio testbed. We used short training symbols (each 0.8 μs) based on the standard-defined short training sequence and conducted experiments by incrementally increasing the number of symbols one by one. As mentioned in Section 3.1, we conducted measurements in an experimental environment with an SNR of 10 dB. Figure 6 shows the packet detection accuracy from the testbed as a function of the PAD preamble length. As illustrated in the figure, when *D* is 2, the detection accuracy reaches 99.985% within 8 μs using a single short training field. Even when *D* is 4, the accuracy remains around 98.2%. For the proposed method, the detection accuracy at 1/2 down-clocking demonstrates performance levels comparable to those reported using recent high-accuracy packet detection methods such as that proposed by Ninkovic et al. [40], with detection accuracies of $10^{-2}$ to $10^{-3}$. At 1/4 down-clocking, the detection accuracy shows a slight reduction but still meets the practical threshold of 90% suggested by Chen et al. [29] for acceptable performance in real-world environments. Based on these experimental results, MILD’s preamble design uses 10 short training symbols (each 0.8 μs, totaling 8 μs) in the PAD preamble.

### 3.4. Design of DAR Preamble

As mentioned in the introduction, we address the preamble length increase from address representation by designing a fixed short DAR preamble using the orthogonal subcarriers of the OFDM symbol. One OFDM symbol contains 52 available subcarriers, allowing up to $2^{52}$ CADDRs to be represented in the DAR preamble at the full clock rate. This large address space ensures that false full-clock recovery due to address sharing does not occur, thereby reducing unnecessary energy consumption. Furthermore, the DAR preamble maintains a fixed length with just one OFDM symbol, making it much shorter than E-MiLi, which increases preamble length to encode address information.

However, utilizing all 52 subcarriers presents technical challenges such as signal aliasing and frequency leakage due to down-clocking. Therefore, we carefully analyze the impact of down-clocking and propose a series of solutions to address these challenges, aiming to maximize the number of subcarriers available for CADDR representation. Finally, we experimentally validate that the false negative probability is low based on a real testbed.

First, we address the signal aliasing issue, previously discussed in Section 3.3. To address aliasing, we modulate binary ‘1’ on only one subcarrier, specifically *D*-1 (where *D* is the down-clocking factor), while modulating binary ‘0’ on the remaining subcarriers. These subcarriers, having no signal power, do not interfere with the folded frequency band, thereby mitigating the signal aliasing problem. The number of usable subcarriers is reduced by a factor of *D*, as only one out of *D* subcarriers is used for CADDR representation.

Additionally, the energy of each OFDM subcarrier can interfere with neighboring subcarriers, a phenomenon commonly known as frequency leakage [16]. This leakage causes variations in signal strength that can reduce the accuracy of the DAR process. A common approach to addressing this issue is to introduce a guard distance *G* between adjacent OFDM subcarriers. By spacing out the subcarriers, the impact of frequency leakage is minimized.

Combining these two solutions, the number of available subcarriers for client address representation is calculated as follows:

(3) $$N_{subcarrier}=\frac{52}{D\times G}$$

According to Equation (3), 13 subcarriers when D=2 and 6 subcarriers when D=4 are available to represent the CADDR binary bits, so the maximum address number $N_{subcarrier}$ is 64 ($2^{6}$) when D=4. This provides a sufficient number of addresses to clients of one AP, even in dense client environments and with a high down-clocking factor.

We evaluate the ability of the proposed DAR process to achieve high detection accuracy with only one OFDM symbol using the USRP/GNURadio testbed. Based on the analysis of the previously considered technical challenges, we determine the number of available subcarriers using Equation (3). The value of *D* is set according to the down-clocking factor, while *G* is fixed at 2 to ensure a guard distance of one subcarrier, thereby mitigating the effects of frequency leakage on adjacent subcarriers. To comprehensively evaluate detection accuracy, we measure two key metrics: the false negative probability and the false positive rate. The false negative probability quantifies the likelihood of the DAR process failing to detect an address during the entire clock recovery process, which directly affects the reliability of packet detection. On the other hand, the false positive rate indicates the frequency of unnecessary wake-up events, which is crucial for energy efficiency, as it leads to unnecessary power consumption.

**Figure 7:**
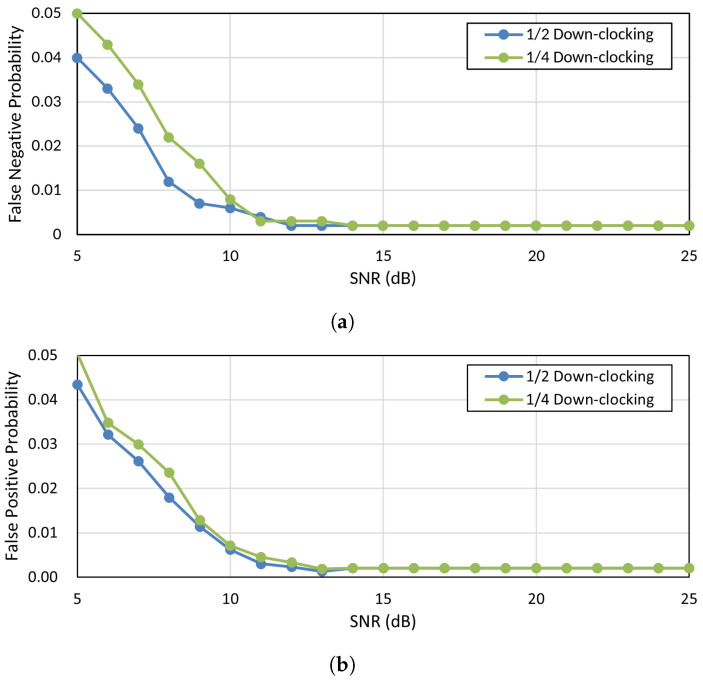
DAR false negative and false positive probability results with USRP testbed. (**a**) DAR false negative probability result with USRP testbed. (**b**) DAR false positive probability result with USRP testbed.

The experimental results are presented in Figure 7, showing the plots of the measured false positive and false negative values across different SNR levels. As illustrated in Figure 7a, the false negative probability consistently remains below 1% when the SNR is greater than or equal to 10 dB. This result demonstrates that the DAR process maintains reliable address detection, even under challenging signal conditions. Similarly, Figure 7b shows that the false positive rate also remains below 1% for SNR values above 10 dB, effectively minimizing unnecessary wake-up events. We choose 10 dB as the benchmark SNR based on Section 3.1, which highlights it as the minimum SNR threshold typically encountered in practical communication environments. Its over 99% accuracy in both false negative probability and false positive rate at this threshold validates the practical feasibility of the proposed approach. These results confirm that the DAR process operates reliably, even in harsh SNR environments, making it suitable for deployment in various real-world scenarios.

The proposed method demonstrates the ability to represent 64 addresses (with D=4) in challenging SNR conditions while achieving high accuracy using only 52 subcarriers within a single OFDM symbol. This high accuracy is attributed to the careful design of the DAR preamble, which accounts for signal aliasing and frequency leakage observed during down-clocking of the client, as discussed in the technical challenges.

The addressing overhead for our method is 4 μs, corresponding to a single OFDM symbol. In contrast, the comparison method, E-MiLi, requires an addressing overhead of 47.8 μs to represent 64 addresses using a preamble-based scheme. Even if E-MiLi reduces energy efficiency by setting the address sharing parameter to 5, the addressing overhead is still 12.6 μs. These results highlight the superiority of our method, as it achieves higher accuracy within a significantly shorter time frame.

## 4. Results

### 4.1. Impact of Preamble Length on Network Throughput

We use the OPNET network simulator to evaluate the network throughput degradation of MILD. To analyze the effect of preamble length, we vary the number of clients connected to the AP from 5 to 50. We generate saturated traffic to measure the maximum throughput. As a comparison, we implemented the traditional A-PSM and E-MiLi [9] in the simulator. For E-MiLi, we simulated both cases with and without address sharing (address sharing factor = 5). The switching delay for clock recovery was assumed to be 9.6 μs [43]. The MILD preamble is modulated by combining one short training field and one OFDM symbol based on the design described in the *PAD Preamble* and *DAR Preamble* sections.

In the baseline method (A-PSM), we set the maximum frame size to 1500 bytes to optimize throughput [44]. Accordingly, with an MPDU size of 1534 bytes, a data rate of 54 Mbps, and a PLCP overhead of 20 μs, the frame duration was calculated to be approximately 247.26 microseconds. We then adjusted this calculated frame duration by incorporating the switching delay and address overhead delay specific to our proposed method and E-MiLi to determine the transmission frame duration in the simulation. Specifically, we applied a 9.6 μs switching delay with an additional 12 μs for MILD, which consists of 8 μs from the PAD preamble (10 short training symbols, each 0.8 μs) and 4 μs from the DAR preamble (one OFDM symbol), and a variable delay depending on the number of addresses for E-MiLi. This configuration facilitates efficient data transmission while preserving fairness, aligning with our goals for optimizing throughput.

**Figure 8:**
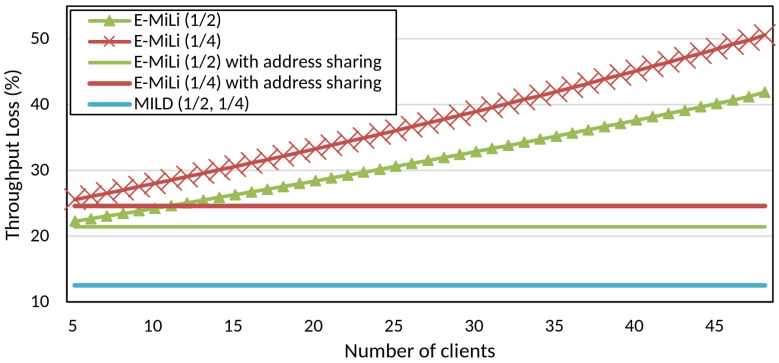
Comparison of throughput loss of E-MiLi and MILD.

Figure 8 illustrates the observed throughput loss under specific down-clocking conditions for both MILD and E-MiLi. In this experimental setup, “1/2” indicates a down-clocking factor D=2, meaning the device’s clock speed is reduced to half of its original speed, while “1/4” represents a down-clocking factor D=4, where the clock speed is reduced to one-fourth of its original speed.

MILD consistently demonstrated a throughput loss of approximately 12.5% for both D=2 and D=4. In contrast, E-MiLi exhibited significantly higher throughput losses, ranging from 21% to 42% for D=2 and 24.6% to 50.5% for D=4. These results clearly indicate MILD’s robustness in maintaining throughput efficiency across varying down-clocking conditions. The enhanced performance of MILD is attributed to differences in address representation mechanisms between MILD and E-MiLi.

The substantial throughput degradation observed in E-MiLi arises from its preamble structure, which increases proportionally with the number of connected clients. As the number of clients grows, the overhead associated with the elongated preamble significantly reduces the airtime available for data transmission, leading to higher throughput losses. For example, E-MiLi requires a duration of 39.5 μs to represent 50 addresses and 12.6 μs to represent 5 addresses.

In contrast, MILD employs fixed-length preambles (PAD and DAR preambles) with a duration of 12 μs, regardless of the down-clocking factor or the number of connected clients. We can represent a large number of addresses with a short OFDM symbol length because OFDM subcarriers are expressed as bits using binary encoding. This DAR preamble design choice minimizes preamble overhead, effectively mitigating the impact on network throughput even as client density increases. Our method supports the representation of up to 8192 addresses at D=2 and 64 addresses at D=4, which is sufficient for typical application environments where a single AP connects to a realistic number of clients. Moreover, even in scenarios exceeding the 64-address limit at D=4, MILD can represent up to 8192 addresses using an additional OFDM symbol duration (4 μs), resulting in a total duration of 16 μs, which does not constitute a considerable overhead.

MILD’s stable throughput performance under diverse down-clocking factors and client densities underscores its suitability for dense network scenarios. By addressing the limitations of variable-length preambles, MILD achieves a balance between energy savings and throughput performance, making it practical for real-world applications.

### 4.2. Energy Efficiency

We evaluated the energy efficiency improvements of MILD using the OPNET network simulator. A-PSM, E-MiLi, and MILD were compared across various traffic patterns. To evaluate the performance across diverse IL distributions, we considered five types of traffic patterns: FTP file transfer, web browsing, voice chat, music streaming, and video streaming. The experiments assume a scenario where 50 clients are uniformly distributed within a 100 m × 100 m building area and connected to an AP. We also account for the increase in fault wake-up occurrences as the clock rate decreases and measure the energy consumption of MILD and E-MiLi under down-clocking conditions where the clock rate is reduced to 1/2 (half) and 1/4 (quarter) of its original value.

**Figure 9:**
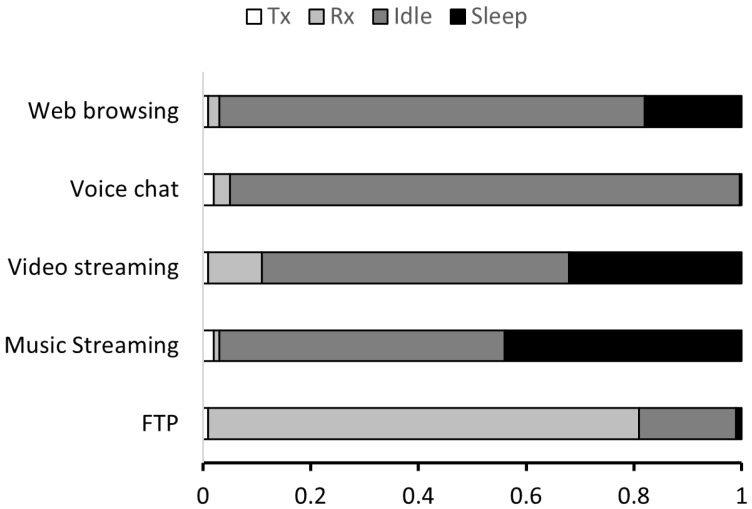
Distribution of Tx, Rx, idle, and sleep states for smartphone devices under various traffic patterns.

Figure 9 shows the proportions of Tx, Rx, idle, and sleep states for smartphone devices under various traffic patterns. In web browsing, the high frequency of interactive traffic between the user and the website results in a high Idle Listening (IL) proportion of approximately 79%. In voice chat, even when there is no active conversation causing Tx or Rx, continuous short traffic prevents the client from entering the sleep state, significantly increasing IL to a maximum of 94.7%. Streaming applications such as video and music streaming exhibit a high concentration of traffic at the beginning of the session. After receiving the necessary data for streaming, the devices spend most of their time in the sleep state. However, IL still occurs due to user interactions and burst traffic at the start of the session, reaching up to 57% and 53%, respectively. In contrast, FTP file transfers generate consistent traffic throughout the session, with only small IL periods observed during the connection initiation and termination phases. Based on this analysis, we implement a real-world traffic generator reflecting application-specific usage patterns and state distributions, which is integrated into the OPNET simulator.

**Figure 10:**
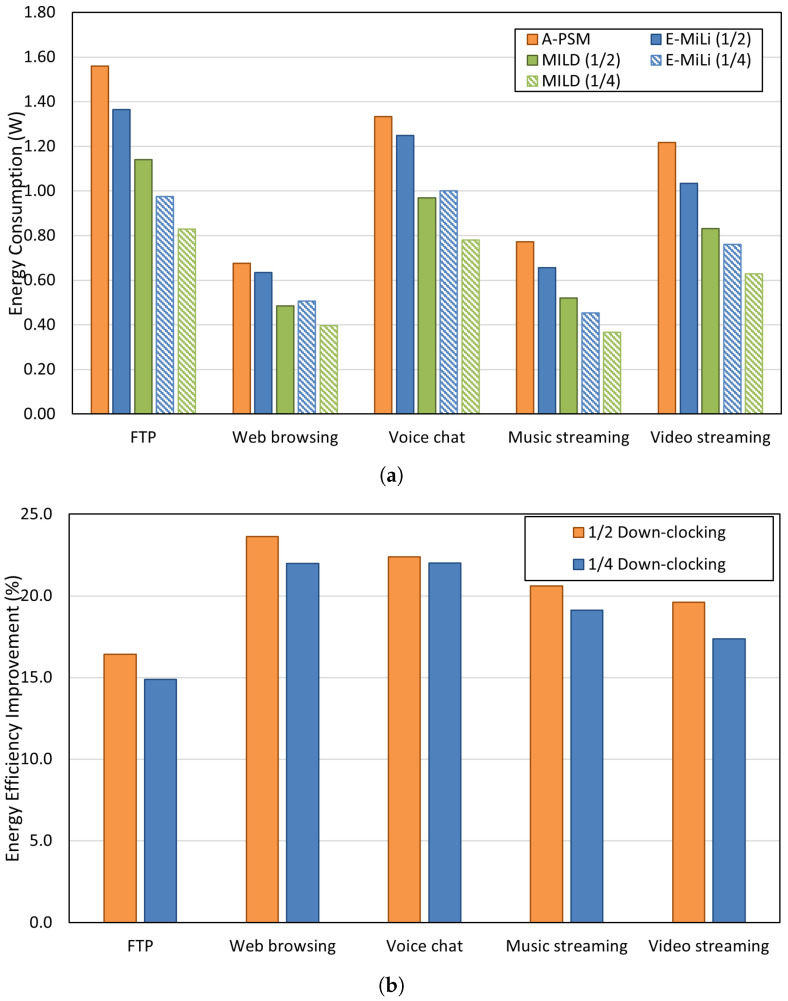
Comparison of energy consumption and efficiency improvement in various traffic patterns. (**a**) Comparison of energy consumption between A-PSM, E-MiLi, and MILD for various traffic patterns, (**b**) Comparison of energy efficiency improvement of MILD over E-MiLi by down-clocking factor.

Figure 10 shows the energy consumption reductions achieved using each method, emphasizing the superior performance of MILD compared to A-PSM and E-MiLi under various traffic conditions. The graph clearly demonstrates the impact of down-clocking on energy savings, showcasing MILD’s efficiency and ability to minimize energy waste. Based on these results, we comprehensively analyze MILD’s energy efficiency improvements for each traffic pattern.

Figure 10a illustrates the energy consumption across different traffic patterns. Down-clocking methods like MILD and E-MiLi exhibit lower energy consumption than the conventional PSM method (A-PSM) because they operate at reduced clock rates during IL periods. Specifically, the energy savings of MILD compared to A-PSM are as follows: 16.79% (1/2 clock rate) and 15.31% (1/4 clock rate) for FTP, 23.81% (1/2) and 21.57% (1/4) for web browsing, 22.40% (1/2) and 22.0% (1/4) for voice chat, 21.21% (1/2) and 17.78% (1/4) for music streaming, and 19.42% (1/2) and 17.11% (1/4) for video streaming. The reduction is relatively higher for web browsing and voice chat, where IL occupies a larger proportion of activity. For music and video streaming, where IL is less dominant, the reduction is slightly lower. In the case of FTP, while energy consumption is relatively high due to prolonged Tx and Rx times, IL during connection/disconnection and contention contributes to moderate energy savings. The slightly lower energy savings observed at the 1/4 clock rate compared to 1/2 can be attributed to reduced PAD and DAR accuracy, which increases fault wake-ups.

We also analyzed MILD’s energy efficiency improvements compared to existing down-clocking methods. Figure 10b shows the energy efficiency gains of MILD over E-MiLi: 16.4% (1/2 clock rate) and 14.9% (1/4 clock rate) for FTP, 23.6% (1/2) and 22.0% (1/4) for web browsing, 22.4% (1/2) and 22.0% (1/4) for voice chat, 20.6% (1/2) and 19.1% (1/4) for music streaming, and 19.6% (1/2) and 17.4% (1/4) for video streaming. As shown in Figure 9, each traffic pattern exhibits unique IL proportions depending on the frequency and flow of data communication. These variations in IL proportions directly influence the frequency of fault wake-ups. Web browsing and voice chat traffic patterns involve short and frequent data exchanges between the client and the AP. This results in a higher proportion of IL compared to Tx and Rx times as the device frequently waits for the next transmission opportunity. While E-MiLi suffers from frequent fault wake-ups and energy waste in such cases, MILD significantly reduces energy consumption through its accurate DAR process. In contrast, music and video streaming traffic exhibit bursty traffic at the beginning of the sessions, leading to lower IL proportions. Although these patterns maintain relatively balanced proportions of Tx, Rx, and IL times, MILD still delivers considerable energy savings due to the overall dominance of IL in the traffic pattern. FTP traffic, characterized by prolonged data reception times and frequent MAC contention, has a relatively lower IL proportion compared to Tx and Rx. As a result, FTP shows the lowest energy efficiency gains among the evaluated traffic patterns.

We performed a numerical calculation to quantify how much the proposed method can reduce overall energy consumption in a practical environment compared to A-PSM and E-MiLi, using the Wi-Fi energy usage proportion data for off-screen states analyzed by Xia et al. [3] and Pramanik et al. [4]. The results show that, compared to A-PSM, the energy savings are as follows: FTP: 9.73% (1/2 clock rate) and 8.88% (1/4 clock rate), web browsing: 13.81% (1/2) and 12.51% (1/4), voice chat: 13.99% (1/2) and 12.76% (1/4), music streaming: 12.33% (1/2) and 10.31% (1/4), video streaming: 11.27% (1/2) and 9.92% (1/4). Compared to E-MiLi, the energy savings are as follows: FTP: 3.71% (1/2) and 8.64% (1/4), web browsing: 13.69% (1/2) and 12.76% (1/4), voice chat: 13.79% (1/2) and 12.76% (1/4), music streaming: 11.95% (1/2) and 11.08% (1/4), video streaming: 11.37% (1/2) and 10.09% (1/4). These results indicate that our proposed MILD method achieves significant energy savings across various usage scenarios, further supporting its effectiveness in minimizing Wi-Fi energy consumption during screen-off states.

These results demonstrate that MILD achieves superior energy efficiency by minimizing fault wake-ups compared to E-MiLi. E-MiLi, while employing address-sharing techniques to reduce preamble overhead, suffers from frequent fault wake-ups due to its design. In contrast, MILD effectively represents a large number of addresses without such trade-offs. Our USRP testbed experiments confirm that MILD’s probability of incorrect clock recovery remains below 1%, even in harsh SNR environments. This accuracy enables MILD to consistently outperform E-MiLi in scenarios dominated by IL, such as web browsing and voice chat. Even in traffic patterns with lower IL proportions, MILD maintains its energy efficiency superiority due to its robust clock management and ability to prevent fault wake-ups.

## 5. Discussion

### 5.1. Compatibility with Existing Wi-Fi Power-Saving Mechanisms

Wi-Fi power-saving mechanisms such as APSD, TWT, and RAW have been introduced to enhance energy efficiency. However, these mechanisms exhibit limited backward compatibility and low adoption rates in commercial devices, posing challenges for widespread deployment. Furthermore, these approaches still experience idle listening issues, leading to unnecessary energy consumption. The proposed MILD technique is not mutually exclusive with APSD, TWT, or RAW but can instead operate alongside these mechanisms. By employing down-clocking, MILD provides additional energy savings during idle listening periods, serving as a complementary enhancement rather than a replacement. Previous studies have also identified idle listening as a persistent challenge in recent power-saving mechanisms. Specifically, APSD suffers from synchronization delays and excessive QoS null frame transmissions, extending idle listening duration. TWT experiences inefficiencies in scheduling and suboptimal wake/doze period negotiations, resulting in increased idle listening. Additionally, RAW, which is designed to function alongside A-PSM, inherits additional idle listening issues due to tail time effects. MILD mitigates these challenges by leveraging OFDM-based packet detection and adaptive down-clocking, effectively reducing idle listening without modifying the MAC operations of APSD, TWT, or RAW.

In addition, APSD, TWT, and RAW encounter significant energy consumption challenges attributed to IL, similar to those observed in A-PSM. Pérez-Costa et al. [13] identified specific scenarios in APSD where IL arises, including synchronization issues and the excessive use of QoS null frames. Yang et al. [14] investigated cases in TWT where improper scheduling or suboptimal wake/doze period negotiation leads to increased IL and energy consumption. Similarly, Kureev et al. [15] analyzed RAW conditions, demonstrating how poorly allocated time windows or device contention exacerbate IL. Notably, RAW, designed to complement A-PSM, introduces additional IL due to the tail time inherent to A-PSM operations. These studies emphasize that IL remains a persistent limitation across power-saving mechanisms, requiring further optimization to mitigate the impact of IL.

Low-power communication technologies such as WUR and Wi-Fi backscatter are primarily designed for IoT and sensor network applications. While these technologies achieve low power consumption, they impose inherent limitations on data rates, hardware requirements, and real-time capabilities. In contrast, MILD is applicable to general communication scenarios, offering higher data throughput, compatibility with existing infrastructure, and minimal hardware dependency, thereby ensuring broader applicability.

Although MILD provides significant energy savings, its direct integration with APSD, TWT, and RAW requires further investigation. Each mechanism possesses unique idle listening patterns, scheduling complexities, and PHY layer dependencies, necessitating a detailed analysis. Moreover, in multi-AP environments, QoS challenges may arise when MILD-enabled clients coexist with non-MILD clients, potentially affecting contention fairness and recovery time for full-clocking. Future research should explore contention-based enhancements, MAC operation optimizations, and PHY layer adaptations to facilitate seamless integration with existing power-saving mechanisms. Addressing these considerations will enable MILD to function as a practical and scalable energy-saving solution, further reinforcing its potential to complement and enhance existing Wi-Fi power-saving technologies.

### 5.2. Operation in Multi-AP Environment

In realistic Wi-Fi communication environments, clients often connect to an AP with better channel conditions or undergo a handoff process due to mobility, connecting to a different AP. To address this issue, two perspectives are discussed for potential future research: the management of client addresses and the differences in on–off operations depending on the use of the MILD technique.

First, the procedure for assigning CADDR during the association between an AP and a client is refined. Specifically, when a client initially connects to an AP, a new mapping between the MAC address and CADDR is created. If the client reconnects to another AP through a handoff (re-association), the existing mapping is deleted, and a new CADDR is assigned by the new AP. These detailed procedures enable MILD to operate stably in environments with multiple APs without address recognition conflicts.

Second, the transmission operations are designed to distinguish between clients and APs that use the MILD technique. During the association process, whether the client uses the MILD technique is verified and stored in the AP’s management table. If the client uses the MILD technique, the AP performs the preamble modulation process defined by MILD. Otherwise, it operates in general A-PSM. Similarly, the client only performs the PAD and DAR processes when connected to an AP that supports MILD operations.

Additionally, environments with multiple APs encounter QoS issues. Specifically, when clients using MILD and those not using it coexist, MILD clients require more time to recover to full-clocking, making them less competitive in contention. To address these QoS challenges, modifications to contention techniques and MAC operations need to be considered.

## 6. Conclusions

In this study, we proposed a novel down-clocking scheme called MILD to reduce energy consumption during idle listening in Wi-Fi communications. MILD introduces a unique preamble design that combines PAD and DAR mechanisms, enabling energy savings without address sharing and addressing throughput degradation issues. The experimental results show that MILD reduces energy consumption by over 20%, particularly excelling in traffic patterns with frequent interactions such as web browsing and voice chat. Additionally, MILD maintained throughput degradation below 12.5% even at high down-clocking rates, outperforming E-MiLi under similar conditions. By maintaining a fixed-length preamble regardless of client count, MILD ensures stable throughput, demonstrating its potential to maximize energy efficiency in diverse network densities and environments. Our study opens up various research directions to support the latest Wi-Fi standards. MILD is not mutually exclusive with APSD, TWT, or RAW, but it can collaborate with these mechanisms. Other power-saving mechanisms also face energy waste caused by idle listening due to factors such as contention or scheduling failures. To address this issue, the proposed approach performs down-clocking during idle listening and utilizes OFDM-based packet detection to enable wake-up. Additionally, it is essential to consider the MAC operations of the power-saving mechanisms supported by each version of the IEEE standard. Additionally, it is necessary to analyze the impact of down-clocking on the PHY headers and the signal processing techniques used during the down-clocking state. With these considerations in mind, we aim to explore more extensive application scenarios in future research.

## Figures and Tables

**Table 1:** Percentage of “Wi-Fi certified” devices that passed WMM-Power Save, Wi-Fi 6, and Wi-Fi HaLow certification programs in November 2022.

Categories	Number of Certified Devices	WMM-Power Save	Wi-Fi 6	Wi-Fi HaLow
AP (home or small offices)	4101	16%	10%	0%
Laptop	1103	37%	26%	0%
Smartphone	19,085	68%	4%	0%
Printer	4015	0%	0%	0%
Smart home	601	0%	0%	0%
TV	10,628	7%	1%	0%

## Data Availability

The data supporting the results presented in this paper are currently not publicly available but may be obtained from the authors upon reasonable request.

## Abbreviations

The following abbreviations are used in this manuscript:

LAN	Local Area Network
IoT	Internet of Things
PSM	Power Saving Mode
A-PSM	Adaptive Power Saving Mode
CAM	Constantly Awake Mode
WNIC	Wireless Network Interface Card
TIM	Traffic Indication Map
STA	Station
WMM	Wi-Fi Multimedia
Tx	Transmission
Rx	Reception
IL	Idle Listening
PAD	Packet Arrival Detection
DAR	Device Address Recognition
AP	Access Point
G	Guard Distance
D	Down-clocking Factor
OFDM	Orthogonal frequency-division multiplexing
DFS	Dynamic Frequency Scaling
USRP	Universal Software Radio Peripheral
CADDR	Client Address
MN	Mobile Node
CSMA	Carrier Sense Multiple Access
CCA	Clear Channel Assessment
BW	Bandwidth
MAC	Medium Access Control
APSD	Automatic Power Save Delivery
TWT	Target Wake Time
RU	Resource Unit
WUR	Wake-Up Receivers
QoS	Quality of Service
OFDMA	Orthogonal Frequency Division Multiple Access
RAW	Restricted Access Window
